# A comparison of linear and nonlinear stability parameters in different clinical forms of multiple sclerosis

**DOI:** 10.1186/s11556-015-0154-7

**Published:** 2015-11-09

**Authors:** Mehrdad Anbarian, Mahnaz Marvi-Esfahani, Mohammad Taghi Karimi, Masoud Etemadifar, Seyed Mohammad Marandi, Mostafa Kamali

**Affiliations:** Faculty of Physical Education and Sport Sciences, Bu Ali Sina University, Hamedan, Iran; Department of Physical Education and Sport Science, Faculty of Humanities, Najafabad Branch, Islamic Azad University, Najafabad, Isfahan Iran; Department of Orthotics and Prosthetics, Faculty of Rehabilitation, Musculoskeletal Research Center, Isfahan University of Medical Sciences, Isfahan, Iran; Department of Neurology, Faculty of Medicine, Isfahan University of Medical Sciences, Isfahan, Iran; Department of Sport Physiology, Faculty of Physical Education and Sport Sciences, University of Isfahan, Isfahan, Iran

**Keywords:** Stability, Multiple sclerosis, Linear, Nonlinear analysis, Spastic, Ataxic, Spastic-ataxic

## Abstract

**Background:**

Multiple sclerosis (MS) is one of the neurological diseases that affect the ability of subjects to stand and walk. The stability of MS subjects has been evaluated in various studies, mostly based on linear approach. Based on this approach it is controversial weather stability of MS subjects differ from normal or not. Therefore, the aim of this study was to evaluate stability in three groups of MS subjects (spastic, ataxic and ataxic-spastic) using both linear and non-linear approaches.

**Method:**

Seventeen healthy and 36 subjects with MS participated in this study. The MS group presenting with spastic, ataxic and ataxic-spastic (each group consisted of 12 subjects) participated in the study. The stability of the subjects was evaluated using Kistler force plate. The difference between stability of the subjects was evaluated using the Multi Analysis of Variance and significant value was set at *P* < 0.05.

**Result:**

There was a significant difference in the mean value of Approximate Entropy (ApEn) in anterior-posterior direction between normal (0.66 ± 0.13) and ataxic (0.85 ± 0.12) and ataxic-spastic (0.90 ± 0.12) subjects (*P* < 0.05) and no difference between normal and spastic groups (0.76 ± 0.13). The results of both linear and nonlinear approaches confirmed that both ataxic and ataxic-spastic subjects had more instability than normal subjects. Although, the mean values of stability parameters increased in spastic compared to normal, the difference was not statistically significant.

**Conclusion:**

Subjects with ataxic and ataxic-spastic MS disorder had difficulty in controlling their stability during quiet standing. The results of this study also confirmed that spasticity of muscles surrounding the hip and knee joints did not influence standing stability in patients with spastic MS.

## Background

Multiple Sclerosis (MS) is one of the most common neurological disorders that affects the ability of subjects during standing and walking [1–4]. The incidence of this disorder differs amongst countries and varies from <5/100,000 inhabitants (low prevalence) to >30/100,000 inhabitants (high prevalence) [5].

Stability is the ability of subjects to return the body from unstable position to a stable position and to keep the body in a stable posture. Research studies have demonstrated that impaired balance is common in people with MS which is not depending on the severity of the disease [1, 3, 6]. Individual with MS have a decreased ability to maintain their position, slow their movement toward limit of stability and have a delay to response to postural perturbations [7]. Frzovic et al. (2000) showed that subjects with MS disorder have weakness in performing clinical tests such as tandem and Romberg tests [8]. The amount of postural disability also depended upon vision [6].

In another study by Yahia et al., it was observed that patients with relapsing-remitting MS and 80% with spastic symptoms have less postural impairments [9]. Contalloub et al. showed that subjects with secondary progressive clinical form of MS have a significant postural impairment [10]. Also, the pyramidal and non-pyramidal MS groups demonstrated concomitant balance impairment in early MS in the absence of clinical disability [3].

MS subjects with low spasticity and high level of spasticity have greater sway than their healthy counterparts but traditional level of significant for subjects with low level spasticity were not reached and there were no differences in anterior-posterior sway range between the groups [11]. Karlon and Achiron, showed that center of pressure (COP) path length, sway velocity and overall sway area increased in subjects with MS [12]. Kanekar et al., used the frequency analysis approach to evaluate balance control in patients with MS. They showed that the mean COP velocity was significantly higher in MS patients than the healthy controls; the magnitude of COP power spectrum in the low frequency band decreased and the medium and frequency band in the medial lateral direction increased in subject with MS [13].

However, the majority of studies on balance have been limited to MS patients without due consideration to the different clinical forms of MS. Moreover, several studies have used traditional linear measures to evaluate balance parameters of patient with MS. Huisinga et al. showed that the mean values of approximate Entropy (ApEn) decrease in patients with MS (Expanded Disability Status scale (EDSS) > 4, with no identified disease symptoms) compared to healthy participants [14]. Negahban et al. used recurrence quantification analysis (RQA), a nonlinear method, to compare the nonlinear dynamic structure of postural sway in patients with MS using three level of postural difficulty. They showed that there was a similar dynamical structure for both the patients and healthy controls and their nonlinear behavior was different under various experimental conditions [15]. Also, Cao et al. estimated EDSS by RQA parameters (percentage of recurrence and mean digital line length) [16].

It is controversial weather stability in patients with MS differs from that of healthy subjects and or depends on the type of the disease. Yahia et al. found that patients with MS with spastic symptoms have no postural impairment [9], these findings are contrary to the results of Sosnof who observed that spasticity in patients with MS increased balance impairment [17]. There are two main approaches for evaluating stability during quiet standing based on COP sways, including linear and nonlinear approaches. In most of the aforementioned studies, stability was evaluated using the linear approach. Based on this method, it is unclear whether stability in patients with MS exhibiting ataxic and spastic symptoms differs from that of healthy subjects. Therefore, the aim of this study was to compare stability in patients with different forms of MS disorders with that of healthy subjects. The main hypothesis of this study was that linear and nonlinear stability parameters in patients with different clinical forms of MS such as spastic, ataxic and a combination of both differ from that of normal subjects.

## Methods

### Subjects

Two groups of normal subjects and MS with cerebellar ataxia, spasticity, spastic with cerebellar ataxia symptoms participated in this study (all participants were female). The MS subjects were selected from those referred to Azahra Hospital for periodic evaluation based on the following inclusion criteria: clinically definite diagnosis of MS, relapse-free during the past 30 days before testing, the range of EDSS:4–6, and between 25 and 52 years old. Healthy subjects were recruited from the staff of Rehabilitation Faculty of Isfahan University of Medical Sciences and were matched with the first group based on age, sex and height. Table 1 shows the characteristics of the subjects participated in this study.

**Table 1:** The characteristics and severity of spasticity of the subjects participated in this study

Parameters	Ataxic G.	Ataxic-Spastic G.	Spastic G.	Normal G.
Age (years)	35.08 ± 8.7	36.08 ± 9.01	39.82 ± 10.22	32.00 ± 7.00
Height (cm)	159.50 ± 4.93	159.83 ± 7.28	160.36 ± 5.61	163.00 ± 0.12
Mass (Kg)	59.42 ± 14.10	52.50 ± 6.20	60.00 ± 11.66	58.00 ± 7.50
EDSS	4.17 ± 1.03	5.17 ± 1.01	4.08 ± 1.10	-
disease duration (year)	8.00 ± 5.58	10.25 ± 5.14	8.73 ± 5.00	-
BARS scale	7.92 ± 2.36	8.60 ± 3.78	1.58 ± 1.08	-
Hip Adductors	0.00 ± 0.00	0.40 ± 0.30	0.83 ± 0.80	-
Knee Extensors	0.12 ± 0.30	1.35 ± 0.97	0.75 ± 0.70	-
Ankle Plantar Flexors	0.33 ± 0.44	2.00 ± 0.10	2.54 ± 0.96	-

Values are expressed as mean ± SD

The *P*-values of difference between the mean values of age, height and mass of normal and MS subjects were 0.07, 0.2 and 0.07, respectively

*EDSS* Expanded Disability Status Scale, *G* group, *BARS* Brief Ataxia Rating Scale

Ethical approval was obtained from Isfahan University of Medical Sciences, Ethics Committee. Consent was obtained from each participant before data collection. Diagnosis of MS was confirmed by a Neurologist using MRI. Patients diagnosed with MS were allocated into three groups based on the results of MRI. If cerebellum, spinal cord, vestibular nerve and the area around vestibular nucleus in brainstem, it was categorized as ataxia. Those with upper motor neurons and corctcospinal fibers impairments were classified as spastic. Ataxic-spastic group consisted of those with involvement in all aforementioned areas [18, 19]. After that, they were stratified according to two clinical examinations using the modified Ashworth Scale for determining of the rate of spasticity and the Brief Ataxia Rating Scale (BARS) for evaluating the ataxia rating.

The severity of disability of the subjects was evaluated using an eight item functional outcome measure (EDSS). This include: motor, sensory, cerebellar, brain stem, visual, mental, sphincters, and others. Each domain is graded from 0 = no disability, to 5 or 6 = maximal disability based on history and physical examination. According to this score 0 was scored as normal and 10 scored as death from MS [20]. Spasticity of lower extremity musculatures was evaluated using a validated scale for clinical assessment of spasticity, the modified Ashworth scale [21]. This involves mobilization of individual joints to provide a clinician-based assessment with an ordinal outcome. The scale ranges from a score of 0 = no increase in tone to 4 = limb rigid in flexion or extension. Three groups of lower extremity musculature were evaluated for the right and left sides (ankle plantar flexor, hip flexor, and hip adductor). The mean values of both right and left sides were recorded for final analysis. Ataxia was scored using Brief Ataxia Rating Scale (BARS) [22].

### Protocol

**Fig. 1:**
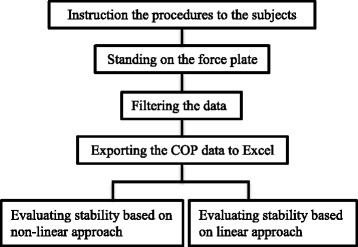
Procedure used to evaluate standing stability in this study

Subjects were instructed about the testing procedures and instruments and then their weight and height were measured and recorded. A Kistler force platform instrumented with piezoelectric force transducers was used to measure the Centre of Pressure (COP) (believed to have a good approximation of sway). They were asked to stand on the force plate for one minute. Data acquired with subjects in double leg stance with feet at pelvic width during normal standing. They were instructed to look straight ahead, with their head erect and their arms at their sides in a comfortable position. Figure 1 shows the protocol used to evaluate stability in this study.

### Parameters

Stability was assessed using both linear and nonlinear approaches based on COP sways. COP excursions in both anteroposterior and mediolateral planes and path length of COP in mediolateral and anteroposterior directions were used as measures of linear method. The following equations, previously described by Karimi et al. were used for analysis [23, 24].

1 $$ \mathrm{COPEAP}\left(\mathrm{mm}\right) = {\mathrm{X}}_{\max }-{X}_{\min } $$

2 $$ \mathrm{COP}\ \mathrm{E}\mathrm{M}\mathrm{L}\ \left(\mathrm{mm}\right) = {\mathrm{Y}}_{\max }-{Y}_{\min } $$

3 $$ \mathrm{PLAP}\ \left(\mathrm{mm}\right)={\displaystyle \sum^{n-1}\sqrt{{\left({x}_{i+1}-{x}_i\right)}^2}} $$

4 $$ \mathrm{PLMLmm}\Big)={\displaystyle \sum^{n-1}\sqrt{{\left({y}_{i+1}-{y}_i\right)}^2}} $$

From the equations above, COPEAP stands for excursion of the center of pressure in the anterior-posterior direction, COPEML is the excursion of the center of pressure in the mediolateral direction, PLAP represents the path length in the anteroposterior direction and PLML is the path length in the mediolateral direction [23]. Nonlinear analysis of COP was performed using the Approximate Entropy (ApEn) parameter as described by Pincus and Kafman [25–27]. ApEn is defined as ApEn (m, r, N), where m stands for the length of compared runs, r is a tolerance and N is the input data points.

The procedure of calculating ApEn is as following [24–26]:

Given a time-series of data u(1), u(2), . . .,u(N) from measurements form a sequence of vectors: x(1), x(2), . . ., x(N - m + 1) in R^m^, defined by x(i) = [u(i), u(i + 1), . . ., u(i + m - 1)].

Define for each *i*, $1\le i\le N - m+1$:

$$ {C}_i^m(r)=\frac{\mathrm{number}\ \mathrm{of}\ j\ \mathrm{such}\ \mathrm{that}\ \mathrm{d}\left[\mathrm{x}\left(\mathrm{i}\right),\ \mathrm{x}\left(\mathrm{j}\right)\right]\le \mathrm{r}}{N - m + 1} $$

Where:

5 $$ \mathrm{d}\left[\mathrm{x}\left(\mathrm{i}\right),\ \mathrm{x}\left(\mathrm{j}\right)\right]= \max\ \left(\left|\mathrm{u}\left(\mathrm{i} + \mathrm{k} - 1\right) - \mathrm{u}\left(\mathrm{j} + \mathrm{k} - 1\right)\right|\right),\mathrm{k}=1,\ 2,\dots, \mathrm{m} $$

6 $$ \mathrm{Define}:\ {\Phi}^{\mathrm{m}}\left(\mathrm{r}\right)=\frac{1}{N - m + 1}{\displaystyle {\sum}_{i=1}^{N\hbox{-} m+1} \log {C}_i^m(r)} $$

Then:

7 $$ \mathrm{ApEn}\left(\mathrm{m},\mathrm{r},\mathrm{N}\right)={\Phi}^{\mathrm{m}}\left(\mathrm{r}\right)\hbox{-} {\Phi}^{\mathrm{m}+1}\left(\mathrm{r}\right) $$

### Statistical analysis

Statistical analysis was carried out using SPSS version 21. The differences in age, height, weight, EDSS, disease duration and BARS scale between four groups with and without MS were determined by multi-analysis of variance (MANOVA). The difference between stability parameters of normal and MS subjects and also three groups of MS participants was evaluated by MANOVA. The relationships between EDSS, BARS scales and Ashworth scale with stability parameters were examined using Spearman Rho correlation. Significance level was set at *P* < 0.05.

## Results

There were no significant differences in age, height and mass between four group and, EDSS and disease duration between three MS groups (*P* > 0.05). The mean value of BARS scale was 7.92 ± 2.36 in ataxic subjects, 8.60 ± 3.78 in ataxic-spastic subjects and 1.58 ± 1.08 in spastic subjects, which confirmed that there was significant difference between spastic group with ataxic and ataxic-spastic subjects.

The mean values of COP excursions in the anterior-posterior direction were 25.65 ± 10.85, 50.05 ± 27.4, 41.68 ± 12.32 and 46.56 ± 15.34 in normal, ataxic, spastic and ataxic-spastic subjects, respectively. There was a significant difference between the mean values of this parameter between normal, ataxic and ataxic-spastic subjects (*P* < 0.05) and no significant difference was found between normal and spastic group (*P* = 0.06). The mean values of it excursion of COP in the mediolateral direction of normal, ataxic and ataxic-spastic subjects were 13.76 ± 5.32 mm, 49.87 ± 50.69 mm and 37.7 ± 25.54 mm, respectively. These were statistically significant different in the mean values of excursion of COP in mediolateral direction between the groups.

The path length of COP in anterior-posterior direction was 460.8 ± 62.6mm for normal subjects compared to 876.3 ± 281.8 for ataxic group (*P* < 0.001). The difference between path length of COP in the mediolateral was significant between normal and ataxic and ataxic-spastic subjects (*P* < 0.05) (Table 2). The range of observed power for balance parameters was 0.88-1.

**Table 2:** Mean and standard deviation of linear approached based on COP sway in four groups

Parameter	Normal G.	Ataxia G.	Spastic G.	Ataxic-spastic G.	*P*-values
COP excursion (mm) (AP)	25.65 ± 10.85	50.05 ± 27.4	41.68 ± 12.32	46.56 ± 15.34	At.&Sp. = 0.6
					At.&A.S. = 0.9
					Sp.&A.S. = 0.9
					No.&At. = 0.002*
					No.& A.S. = 0.01*
					No.&Sp. = 0.06
COP excursion (mm) (ML)	13.76 ± 5.32	49.87 ± 50.69	31.31 ± 21.51	37.7 ± 25.54	At.& Sp. = 0.4
					At.&A.S. = 0.7
					Sp.&A.S. = 0.9
					No.&At. = 0.009*
					No.& A.S. = 0.1
					No& Sp. = 0.3
Sum path length (mm) (AP)	460.8 ± 62.6	876.3 ± 281.8	634.2 ± 160.6	950.7 ± 358.1	At.& Sp. = 0.06
					At.&A.S. = 0.8
					Sp.&A.S. = 0.008*
					No.&At. = 0.000*
					No.& A.S. = 0.00*
					No& Sp. = 0.2
Sum path length (mm) (ML)	502.2 ± 77.2	978.2 ± 463.3	678.4 ± 264.2	1014.4 ± 411.2	At. & Sp. = 0.1
					At. &A.S. = 0.9
					Sp. &A.S. = 0.06
					No.&At. = 0.002*
					No.&A.S. = 0.001*
					No& Sp. = 0.4

Values are expressed as mean ± SD, *P*-value less than 0.05 (95% confidence interval), *P < 0.05

At.: ataxic group; Sp.: spastic group; A.S.: ataxic-spastic group; &: *P*-value between 2 groups

*COP* Center Of Pressure, *AP* Anterior-Posterior, *ML* Medio-Lateral

Nonlinear analysis was also carried out in this research study. The mean value of ApEn in anterior-posterior direction was 0.66 ± 0.13 in normal, 0.85 ± 0.12 in ataxic, 0.76 ± 0.13 in spastic and 0.90 ± 0.12 in ataxic-spastic subjects. There was a significant difference between the mean values of these parameters between normal, ataxic and ataxic-spastic subjects. The mean value of ApEn in the mediolateral was significantly higher in ataxic, ataxic-spastic compared to the healthy subjects. There was no significant difference in ApEn in ML and AP directions between normal and subjects with spastic MS (*P* > 0.05). Table 3 shows the mean value of stability parameters, based on non-linear approach.

**Table 3:** Mean and standard deviation of nonlinear approached based on COP sway in groups

Parameter	Normal G.	Ataxia G.	Spastic G.	Ataxic-spastic G.	*P*-values
Approximate Entropy (AP)	0.66 ± 0.13	0.85 ± 0.12	0.76 ± 0.13	0.90 ± 0.12	At.&Sp. = 0.3
					At.&A.S. = 0.8
					Sp.&A.S. = 0.03*
					No.&At. = 0.001*
					No.&A.S. = 0.00*
					No.&Sp. = 0.1
Approximate Entropy (ML)	0.67 ± 0.15	0.89 ± 0.18	0.77 ± 0.18	0.93 ± 0.15	At.& Sp. = 0.3
					At.&A.S. = 0.9
					Sp.&A.S. = 0.09
					No.&At. = 0.003*
					No.&A.S. = 0.00*
					No& Sp. = 0.2

Values are expressed as mean ± SD, *P*-value less than 0.05 (95% confidence interval), *P < 0.05

*AP* Anterior-Posterior, *ML* Medio-Lateral

**Fig. 2:**
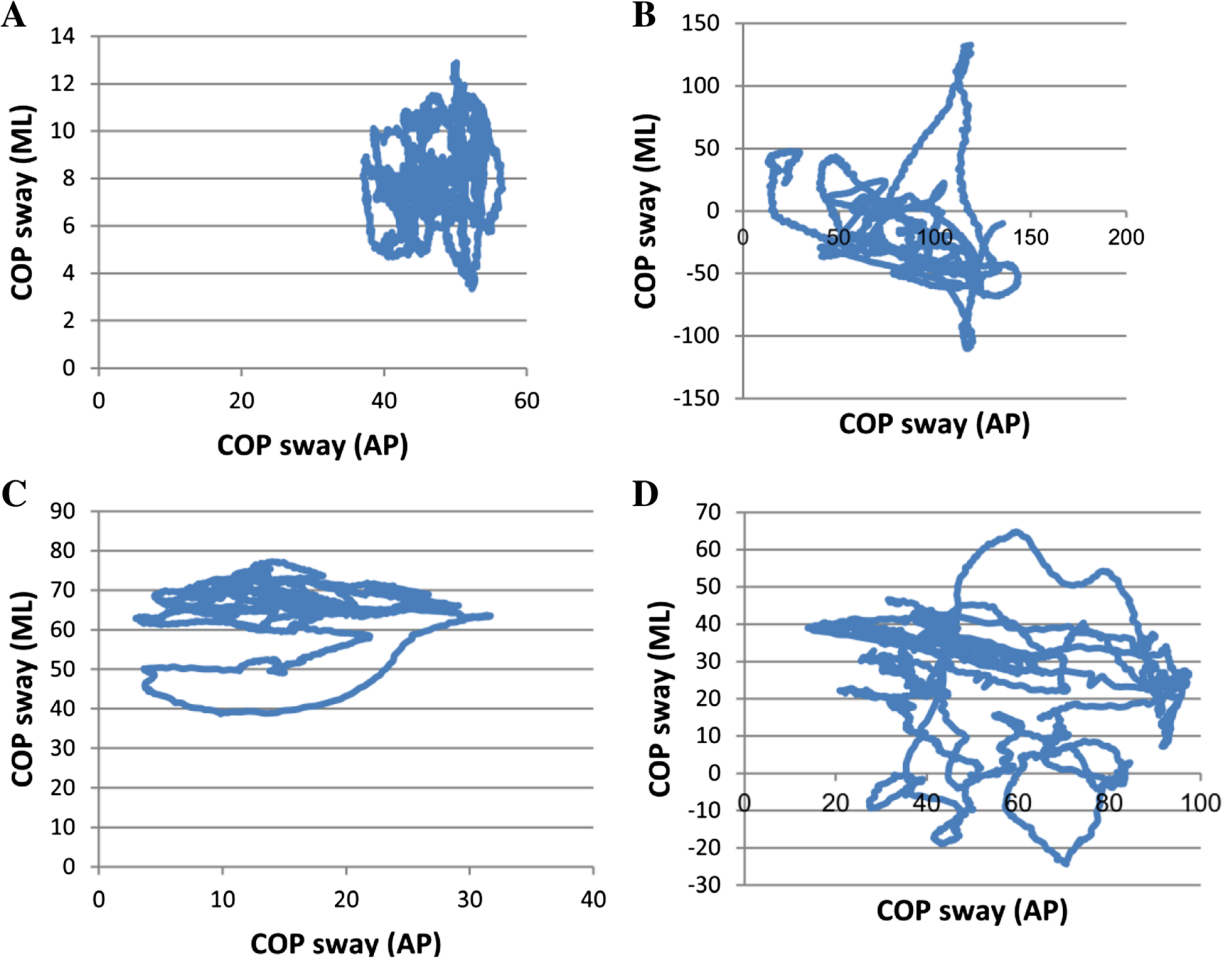
Representation of COP of a control subject (**a**), a person with Ataxia (**b**), a subject with spastic (**c**) and a person with Ataxic-Spastic (**d**)

Figure 2 shows the pattern of COP sway in normal, spastic, ataxic and ataxic-spastic subjects.

It can be seen in the Figure those three individuals with MS have greater sway than the control subject.

The mean value of spasticity of hip adductor was 0.00 ± 0.00, 0.40 ± 0.30 and 0.83 ± 0.80 in ataxic, ataxic-spastic and spastic groups, respectively. The spasticity of ankle plantar flexor bared on Ashworth Scale was 0.33 ± 0.44 in ataxic, 2.00 ± 0.10 in ataxic-spastic and 2.54 ± 0.96 in spastic group (Table 1).

The relationship between EDSS, BARS scale and Ashworth scale with stability parameters is shown in Table 4. As it can be seen from this Table, there was a significant correlation between two parameters of stability and the mean value of EDSS. The correlation between spasticity and stability parameters was not significant. Also, there was a significant correlation between four parameters of stability and the mean value of BARS.

**Table 4:** The correlation and *P*-value between EDSS, Ashworth scale and BARS scale and stability parameters

Parameters	Excursion COP (AP)	Excursion COP (ML)	Path length COP (AP)	Path length COP (ML)	Approximate Entropy (AP)	Approximate Entropy (ML)
EDSS	0.178	0.248	0.435	0.412	0.199	0.412
*P* -values	0.15	0.07	*0.01	*0.01	0.12	0.13
Ashworth	−0.145	−0.179	−0.094	−0.115	−0.037	0.029
*P*- values	0.20	0.14	0.29	0.25	0.41	0.43
BARS	0.259	0.487	0.610	0.578	0.330	−0.210
*P*- values	0.11	*0.008	*0.001	*0.002	*0.05	0.16

Values are expressed as mean ± SD, *P*-value less than 0.05 (95% confidence interval), *P < 0.05

*AP* Anterior-Posterior, *ML* Medio-Lateral

## Discussion

Subjects with Multiple Sclerosis (MS) have decreased ability to maintain their posture. Based on the findings of various studies, it is unclear whether stability of MS subjects differs from that of healthy subjects. Moreover, it is not clear whether the type of MS influence their stability or not. Therefore, this study compared stability of patients with different forms of MS disorder with that of healthy subjects.

As it can be seen from the results of this study, the stability of MS subjects (ataxic and ataxic-spastic) differs significantly from that of normal subjects. Both linear and nonlinear parameters confirmed that their stability decreased in both anteroposterior and mediolateral directions. But the subjects with spastic symptoms have less postural impairments than the ataxic and ataxic-spastic groups. In addition, there was no difference in linear and nonlinear balance parameters between healthy and spastic subjects.

Within the current study, patients with spasticity symptoms especially in plantar flexor muscles used hip and trunk strategies to control their posture, so that their balance increased but not significantly. The results of this study confirmed the findings of Yahia et al. [9] and also the results of study of Sosnof et al. which showed that stability in subjects with low spasticity was similar to that of healthy subjects and those with high level spasticity [11]. In another study, Sosnof mentioned that the stability of ataxic MS subjects decreased due to weakness of muscles surrounding the ankle, knee and hip joints [17]. Yahia et al. showed that there was a positive correlation between muscle strength and balance parameters especially for hamstring muscles [9].

There is only one study that has used the non-linear approach to evaluate standing stability. In the study by Huisinga et al., the mean values of ApEn decreased in MS subjects compared to healthy participants but they did not identify the disease symptoms. They concluded that the adaptability of MS subjects to perturbation decreased [14]. However, in the current study, the ApEn increased significantly especially in ataxic and ataxic-spastic subjects. Both the increase and decrease in the value of ApEn represents instability. However, ApEn increased in subjects with muscular weakness. In contrast it decreased in subjects with high spasticity and rigidity. The results of correlation did not support the finding of Sosnoff et al. (2010) regarding instability of MS subjects with high degree of spasticity [11].

Based on the results of this study, the correlation between most of stability parameters and EDSS was significant. This means that those with more impairment may be more unstable to control their standing balance. The reason for this is not known, however, it may be due to muscles weakness which cannot control the motion of COM efficiency.

There are some limitations to this study, which should be acknowledged. Only stability during quiet standing was analyzed in this study. As result it difficult to judge if dynamic stability is also impaired in these subjects using these approaches or not. Another limitation, is that the sample sizes for the different MS groups were small, which may limit the generalizability of the findings. It is hoped that these limitations can be addressed in future studies.

## Conclusion

Stability of subjects with various kinds of MS disorders (Ataxi, Spastic and Ataxic-spastic) differed from that of healthy subjects, this may be due to muscle weakness but it differed in spastic subjects with high EDSS. Although the association between instability and spasticity was supported in the previous studies, the results of this study did not support it.
